# Pharmacodynamic analysis of an agonistic antibody to the costimulatory receptor GITR

**DOI:** 10.1186/2051-1426-3-S2-P192

**Published:** 2015-11-04

**Authors:** Gordon Moody, Jodi Moriguchi, Ji-Rong Sun, Patricia McElroy, Hong Tan, Yannick Bulliard, Beltran Pedro

**Affiliations:** 1 Amgen, Inc., Thousand Oaks, CA, USA

## 

GITR/TNFRSF18 is a member of the TNF-receptor superfamily preferentially expressed on regulatory T cells (Tregs) and activated T effector cells. Antibody agonists to GITR claim two distinct mechanisms to overcome the repressive tumor microenvironment and drive anti-tumor efficacy *in vivo*: receptor agonism (forward signaling) on T effector cells and FcγR-mediated Treg depletion. We sought to better understand the contribution of these two mechanisms using pharmacodynamic readouts relating target coverage, Treg depletion and efficacy using isotypic variants of a surrogate antibody against mouse GITR, DTA-1.

First, target coverage was determined in spleen, tumor and draining lymph node following treatment with a single dose of mouse IgG2a DTA-1. In this study, efficacy correlated with doses that covered >90% GITR-expressing intratumoral leukocytes and depleted >90% intratumoral Tregs at 24 hours post-dose. Though displaying equivalent agonistic activity *in vitro* and achieving a similar level of target coverage, the mouse IgG1 N297A variant of DTA-1 neither depleted Tregs nor displayed anti-tumor activity *in vivo*, in confirmation of recent literature. To further explore the influence of Fc engagement, additional DTA-1 isotypic variants were generated and tested *in vivo*. In this study, we confirmed that preferential engagement of Fcγ receptors was necessary for optimal activity, as the mouse IgG1 DTA-1 variant failed to regress tumors. Additionally, we identified a variant with enhanced Treg depletion properties, however, the enhanced depletion did not translate to improved anti-tumor efficacy.

Lastly, we sought to understand if mouse IgG2a DTA-1-would enhance the effect of PD-1 / PD-L1 blockade *in vivo*. Using the MC38 tumor model, we observed synergistic tumor regression in the combination group versus either monotherapy. Given the likely non-overlapping mechanism of the antibodies, the results suggest that an ADCC-enabled agonistic GITR antibody could provide benefit to human cancer patients in combination with, or refractory to, PD-1/PD-L1 inhibitors

